# Effect of Temporal Organization of the Visuo-Locomotor Coupling on the Predictive Steering

**DOI:** 10.3389/fpsyg.2012.00239

**Published:** 2012-07-11

**Authors:** Yves Philippe Rybarczyk, Daniel Mestre

**Affiliations:** ^1^Department of Electrotechnical Engineering, New University of LisbonLisbon, Portugal; ^2^Institute of Movement Sciences, CNRS and University of the MediterraneanMarseille, France

**Keywords:** visuo-locomotor coupling, temporal coordination, anticipation, 2/3 Power Law, path formation, robotics

## Abstract

Studies on the direction of a driver’s gaze while taking a bend show that the individual looks toward the tangent-point of the inside curve. Mathematically, the direction of this point in relation to the car enables the driver to predict the curvature of the road. In the same way, when a person walking in the street turns a corner, his/her gaze anticipates the rotation of the body. A current explanation for the visuo-motor anticipation over the locomotion would be that the brain, involved in a steering behavior, executes an internal model of the trajectory that anticipates the completion of the path, and not the contrary. This paper proposes to test this hypothesis by studying the effect of an artificial manipulation of the visuo-locomotor coupling on the trajectory prediction. In this experiment, subjects remotely control a mobile robot with a pan-tilt camera. This experimental paradigm is chosen to manipulate in an easy and precise way the temporal organization of the visuo-locomotor coupling. The results show that only the visuo-locomotor coupling organized from the visual sensor to the locomotor organs enables (i) a significant smoothness of the trajectory and (ii) a velocity-curvature relationship that follows the “2/3 Power Law.” These findings are consistent with the theory of an anticipatory construction of an internal model of the trajectory. This mental representation used by the brain as a forward prediction of the formation of the path seems conditioned by the motor program. The overall results are discussed in terms of the sensorimotor scheme bases of the predictive coding.

## Introduction

Various studies showed that many different human movements seem to follow a same mathematical relationship known as the “2/3 Power Law” (Lacquaniti et al., 1983; Viviani and Schneider, 1991). So, whether the action is writing (Viviani and Cenzato, 1985) or walking (Vieilledent et al., 2001), an identical constraint relationship between the velocity and the curvature of the motor trajectory is involved. This law states that the angular velocity of the end effector is proportional to the two-thirds root of its curvature or, equivalently, that the instantaneous tangential velocity is proportional to the third root of the radius of curvature. It means that the velocity of the movement decreases in the highly curved parts of the trajectory and increases when the trajectory becomes straighter. The hand or the steps slow down near regions of high curvature presumably to ensure an accurate movement. In fact, if you ask someone to follow an elliptic path, the sectors where the curvature radius is the smallest induce the largest precision error. What is most remarkable is the correlation between these high inflection parts and the number of occurrences of ocular fixations (Reina and Schwartz, 2003). This observation presumes that the motor control would need a superior visual feedback to compensate the higher instantaneous complexity of the geometry of the movement.

**Figure 1:**
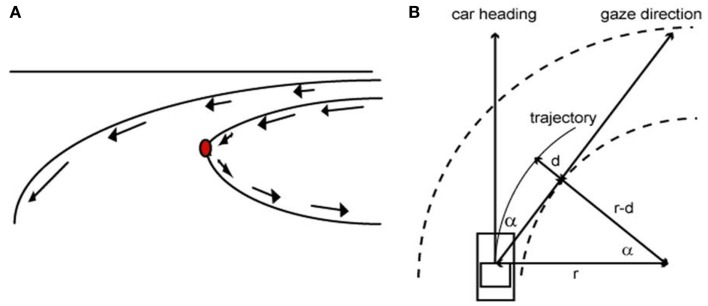
**(A)** The tangent-point of the inside curve is visually immobile in the dynamic visual field of the observer. **(B)** When the driver’s trajectory follows the bend, looking toward the tangent-point provides a prediction of the road curvature (after simplification, 1/*r* = α2/2*d*).

Studies carried out on the steering behavior show that the ocular fixation on the point of maximal curvature does not only occur for the visually controlled manual guidance (Land, 1998; Chattington et al., 2007). Land and colleagues have shown that automobile drivers fixate on a similar point near the tangent of the road ahead whether they are familiar with the road (Land and Tatler, 2001) or not (Land and Lee, 1994). For example, Land and Lee (1994) demonstrated that, while driving an automobile, humans invariably fixate their eyes near the point of maximum curvature, along the road ahead, approximately 1–2 s prior to reaching the curve and maintain their gaze relatively fixed on that point while bending. The reason why the driver looks toward the tangent-point of the inside curve is because of the singular optical properties of this part of the road. Indeed, at this specific point there is a reversion of one of the components of the optical flow, which maintains an identical position in the visual field for a constant curvature and, consequently, makes this location particularly relevant to stabilize the vehicle’s trajectory (Kandil et al., 2009). A simple mathematic relationship shows that this stable cue can be used by the driver to predict the curvature of the road (Figure 1).

Other studies show a similar pattern of visual anticipation over motion during the visual guidance of the human locomotion (Grasso et al., 1996; Hollands et al., 2002). Thus, when an individual completes a curvilinear movement, his/her gaze axis is rotated in relation to the walking direction in such a way that the direction of the gaze points toward the inside of the trajectory. In the same way, if the task consists in turning a corner, the subject’s eyes orient to the obstacle before the walker reaches it (Grasso et al., 1998). The visual axis only realigns with the rest of the body once the obstacle is overcome. Therefore, the visual guidance of the locomotion seems organized according to a visuo-locomotor coupling that initiates in the cephalic organ and finishes in the pelvic members. In other words, the strategy used by the human being is not “I look where I go” but “I go where I look.”

A consensual explanation for the visuo-motor anticipation over the locomotion would be that the brain, involved in a steering behavior, executes an internal model of the trajectory that anticipates the completion of the path, and not the contrary (Berthoz, 1997). For any visually guided actions, this model implements proactive eye movements which are crucial for planning and controlling (Land et al., 1999; Land and McLeod, 2000; Johansson et al., 2001). The motor bases of the visual perception are extensively studied in order to understand the capabilities of the human being to predict the ongoing actions of him/herself and conspecifics (Flanagan and Johansson, 2003; Friston et al., 2011; Springer et al., 2011). According to this theory, it seems that the individual uses his/her own motor repertoire to run internal sensorimotor simulations that predict the future course of executed and/or observed actions (Wilson and Knoblich, 2005; Schubotz, 2007; Sebanz and Knoblich, 2009, for reviews).

All in all, the visuo-motor system implements a predictive coding in order to compensate the time the nervous system takes to percept, process, and respond to a stimulus in the environment. In everyday life situations such as a visual tracking of a car, for instance, it allows us to know when we can cross the road safely. If the ocular pursuit – which is a quite slow system (less than 100°/s) – did not make predictions on the future trajectory of dynamic artifacts, the motion of many daily stimuli, like the car, would be too fast to be tracked in real time. Actually, it is not the moving target that is tracked, but an internal simulation of the predicted trajectory of the target (Yasui and Young, 1975; Berthoz, 1997). Prediction is also crucial in catching an object in motion, such as a ball. Lacquaniti and Maioli (1989) showed that such action involves an internal model, or representation, of the expected dynamic interactions during the impact, which is constructed on the basis of *a priori* knowledge and the sensorial inputs (ball and body velocity). The internal model response to the ball input is compared to the arm movement, which generates an error signal. This signal is used to update the motor control and representation. When those dynamic interactions between the ball and the limb are correctly predicted, the unfolding body movement to catch the ball will be described in terms of a smooth arm trajectory that follows the “2/3 Power Law.”

Although motor representations seem clearly involved in predicting the action, there is no direct investigation about the impact of the manipulation of natural motor schemes on motion prediction. So, the present article aims to study whether the temporal organization of the action influences the predictive curvature of the upcoming trajectory. The quality of the path prediction is gaged through the analysis of the kinematic characteristics of the movement. More precisely, we used two fundamental parameters of the motion, being (i) the number of occurrences of each radius of curvature and (ii) the relationship between the speed and the curvature of the trajectories. In order to complete the study, we applied an experimental paradigm that enables us to manipulate the coupling sequence between the visual receptor and the effector organs.

Considering the fact that we cannot directly alter the head-body coordination of the human being, we worked with a mobile robotic device which is remotely controlled by the subject. In this situation, the coupling between the embarked pan-tilt camera and the mobile platform can be easily and precisely manipulated. Consequently, the data are not related to the direct recording of the human movement but actually to the performance of an individual in a condition of teleoperation. Although it is an indirect measurement, many studies on the interaction between a human operator (Rybarczyk et al., 2002; Cardinali et al., 2009; Carlson et al., 2010; Rybarczyk and Mestre, 2011) or an animal (Iriki et al., 1996, 2001) and simple tools or robotic devices show evident proofs of an assimilation of the artifact in the corporal schema of the user, in a such way that the two entities become united in a single one (Maravita and Iriki, 2004; Rybarczyk et al., 2012, for reviews).

## Materials and Methods

**Figure 2:**
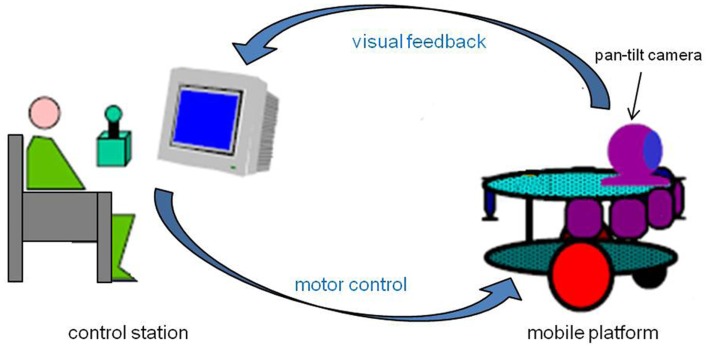
**Experimental configuration**. The subject is seated at the control station from which s/he can remotely control the robotic device and receive a visual feedback from the pan-tilt camera embarked on the mobile platform.

The telerobotic system used in the experiment is constituted by two main components: a mobile platform and a control station (Figure 2). The robotic platform is equipped with a mobile camera, which enables a motion control according to the pan plan. The robot is moved by two independent steering wheels and a free wheel in front of the vehicle allowing its stability. The engines are the same type as those which equip electric wheelchairs. The optical camera’s view field is 50° in the horizontal and 38° in the vertical dimension. This sensor “sends” to the operator an image of the environment in which the robot evolves, on a terminal display 23 cm in height and 31 cm in width. The whole system, engines, and sensors, is controlled by a PC embarked on the robot. This PC is connected to the computer of the control station through a TCP/IP HF connection. Client/server software architecture structures the computing part. The control interface is using the PC keyboard, through which the operator controls the direction and displacement velocity of the platform. For security reasons, the maximum speed of the robot was limited to 1 m/s.

**Figure 3:**
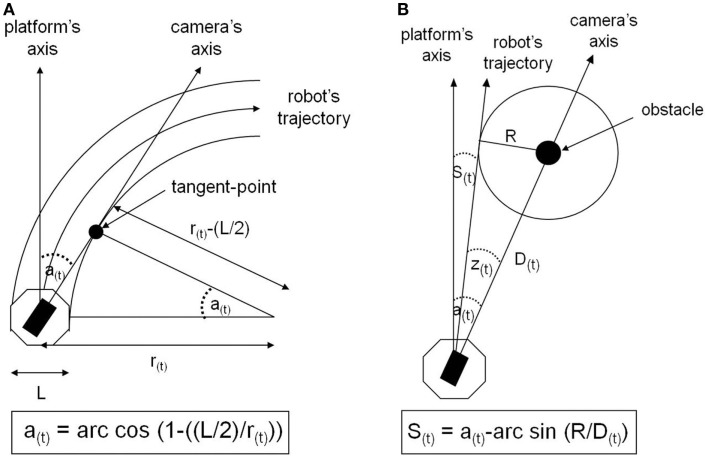
**(A)** Implementation of visuo-motor anticipation according to a non-human-like model. The camera’s rotation angle is computed by the curve radius (*r*) of the robot’s trajectory, using trigonometric laws. Here, *cos a* = (*r*(*L*/2))/*r*, where the semi-width of the robot equals *L*/2. The radius (*r*) is obtained by dividing the translation velocity by the rotation velocity of the robot. **(B)** Implementation of visuo-motor anticipation according to a human-like model. The robot’s navigation angle (*S*) is defined as the difference between the angle (*a*), between the direction of the camera and the heading of the robot, and the angle (*z*), between the direction of the camera and the tangent to the orbit of safety (*R*). This angle z is calculated by using trigonometric rules in such a way that *sin z*(*t*) = *R/D*(*t*)*. D*, the distance between the robot and the landmark, is obtained by the ratio of the rate of change of the camera angle to velocity, such as *D* = (*v/*(*da/dt*)·*sin a*.

**Figure 4:**
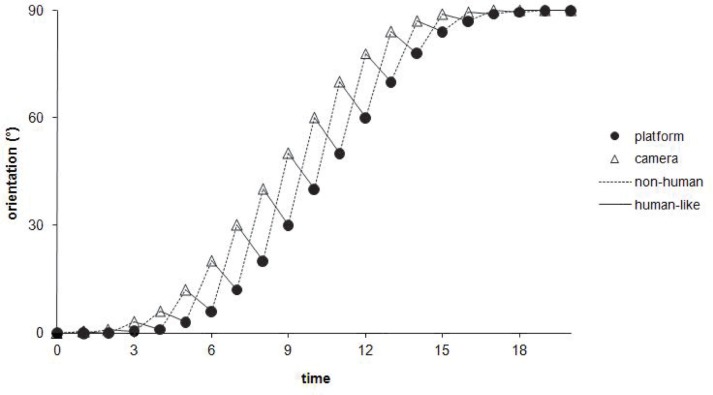
**Theoretical representation of the course, through time, of the angular orientation for the mobile platform and the camera, during a path curvature of 90°**. It is important to notice that, at an instantaneous time *t*, the camera’s angle (open triangles) is always superior to the platform’s angle (filled circles), whatever the model implemented – except, of course, for the “control condition” (not represented in the figure) in which the angular position is aligned with the platform. However, the two models of anticipation differ from each other regarding the temporal hierarchy of the visuo-locomotor coupling’s organization. The angular anticipation of the camera is set at *t* + 1 in relation to the platform, in the “non-human model” (dotted lines), whereas this camera’s angle leads at *t* − 1 the future position of the robot, in the “human-like model” (continuous lines).

Three independent groups of seven subjects carried out one of three experimental conditions of visuo-motor anticipation. The first situation is a “control” condition, in which there is no anticipation, since the camera is motionless, aligned with the orientation of the robot. In the second condition, called “non-human,” the temporal organization of the visuo-locomotor coupling is reversed regarding the human natural motor scheme. Although the camera visually anticipates the platform’s displacement, this anticipation is only reactive, because it depends on the orders the operator sends effectively to the mobile platform. Thus, the angular anticipation of the camera is a simple consequence of the robot’s changes of direction (Figure 3A). The third condition is called “human-like,” because the camera-platform coupling is implemented exactly according to the temporal organization of the human visuo-locomotor coupling. This means that the operator actively controls the camera orientation and, then, the direction of the robot is automatically computed following the azimuth angle of the visual referential (Figure 3B). In short, the difference between the experimental conditions is based on the temporal hierarchy of the visual sensor’s reorientation in relation to the locomotor organs (Figure 4). In terms of timing, the camera deviates 1 s ahead in relation to the platform, for the “human model,” whereas it deviates 1 s later, for the “non-human model.”

**Figure 5:**
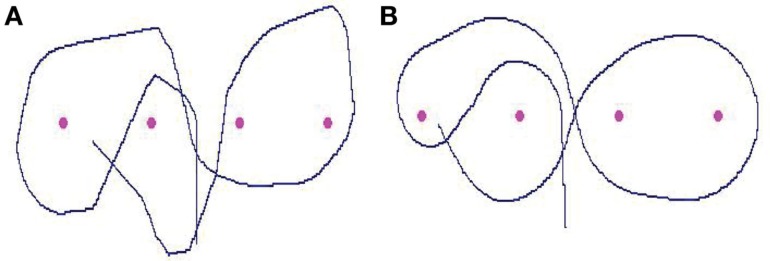
**(A)** Representation of a sharp trajectory carried out by the robot during the slalom task between the four landmarks. This sharp pattern – related to the stops that happen before the main changes in the steering trajectory – is characteristic of a situation in which the camera is maintained fixed, pointing in the same direction of the vehicle’s axis. **(B)** Representation of a smooth (or curvilinear) trajectory carried out by the robot during the slalom task between the landmarks. This uniformly smoothed pattern is a typical example of a condition in which the camera visually anticipates the platform displacement.

In the three conditions, the subjects are placed in a teleoperated situation, i.e., they only have an indirect visual perception of the experimental environment. It is important to notice that the front part of the platform is visible from the camera’s view field, in order to provide the participant with visual information about the angular rotation of the camera in relation to the robot’s heading. An initial training stage is carried out by the subjects, in which they learn how to control the robot. This training lasts about 30 min. After this period, none of the participants reported having difficulty in driving the robot. The training is completed in a different experimental environment than that during the recorded experiment. In order to analyze a pure predictive behavior, which must not be contaminated by the learning environment (Péruch and Mestre, 1999), each subject only performs one attempt. The task consists in making the robot do a slalom course between four boundary marks (Figure 5). The path to be followed is always the same. The instructions given to the subjects consist in carrying out the course as fast as possible without colliding with the boundary marks. The analysis of the results is carried out according to two parameters: the smoothness (minimum jerk) of the trajectories and the relationship between the geometry and the kinematics of the robot’s trajectory (“2/3 Power Law”).

## Results

The first analysis of the movement’s kinematics is about the jerk in the control of the robot’s trajectories. One way to quantify the path’s smoothness is to calculate the instantaneous radius of curvature of each trajectory, then to evaluate the distribution frequency of the radius for all trials (Péruch and Mestre, 1999). More specifically, the curve radius (*r*) is computed from the instantaneous linear velocity (*v*) divided by the instantaneous rotation speed (*w*), according to the following equation:

$$\begin{array}{rcll} r\left(\text{m}\right)=\frac{v\left(\text{m}∕s\right)}{w\left(\text{radians}∕s\right)} & & & \end{array}$$

Afterward, the curve radius is converted into a decimal logarithm. Thus, if the robot has a low linear velocity and a high rotation speed, the curve radius will be very small (<1), and will get smaller as the rotation speed increases. The logarithmic value of *r* will be consequently negative. Conversely, if the robot combines a translation and a rotation (curvilinear trajectory), the radius of curvature will be high (≥1) and its logarithm will be superior or equal to zero. A trajectory in which the participant stops and makes a single rotation gives a bimodal distribution of the curve radii, with one spike centered on negative values of the logarithm and another spike centered on positive or null values. On the contrary, a smooth (or curvilinear) trajectory will be identified by a unimodal pattern of distribution centered on a value higher or equal to zero of the logarithm of the curve radius. For each trajectory, the distribution of the logarithm of the curve radii is computed and distributed in 15 categories. These categories represent contained values between −4 and −3.5, −3.5 and −3, −3 and −2.5, …, 2.5 and 3, i.e., according to a constant scale of ranges that allows us to analyze the results from an ANOVA test. Finally, the distributions are normalized, with the occurrences of curve radii in each category being expressed as a percentage of the total number of occurrences for each trajectory.

**Figure 6:**
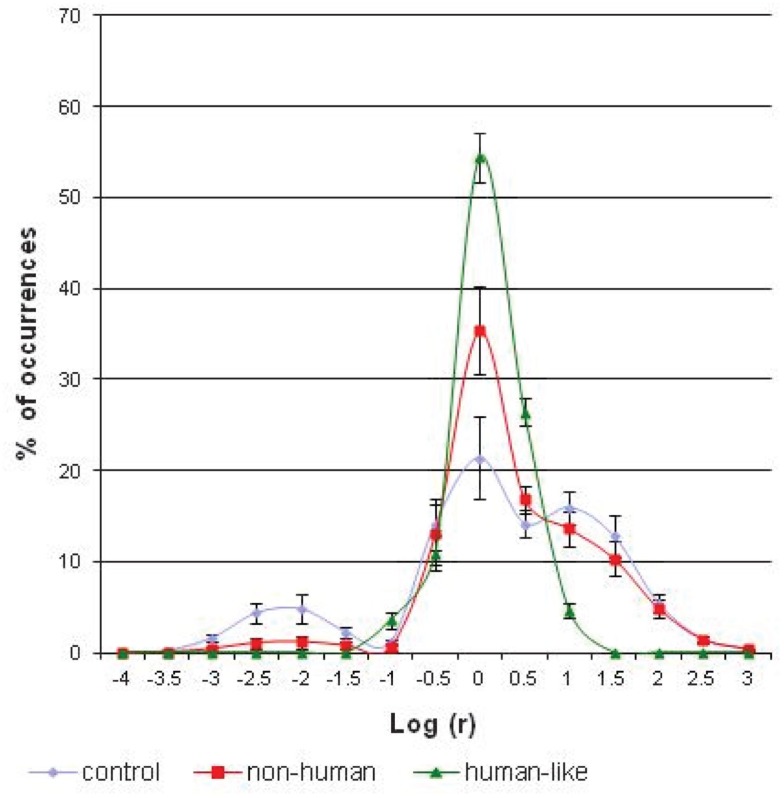
**The average distribution of (logarithms of) curve radii, expressed as a percentage of the total number of occurrences, following the three experimental conditions of anticipation**. The percentage of occurrences of the smallest spike of curve radius (around −2) is significantly lower (*p* < 0.04) while the largest spike of curve radius (around 0) is significantly higher (*p* < 0.0006) in the “human-like” mode of control, as compared with the two other robot modes of control.

The results indicate a significant effect of interaction between the visuo-motor conditions and the category factor [*F*(28; 252) = 21.28; *p* < 0.00001]. Figure 6 shows that the percentage of occurrences of small and large curve radii is different according to the experimental condition. The largest spike, corresponding to the curvilinear trajectories, is significantly higher in the conditions of visual anticipation than in the condition without anticipation [*F*(2; 18) = 28.61; *p* < 0.00001]. Conversely, the smallest spike, corresponding to the single rotations, is statistically lower in the conditions of anticipation than in the control condition [*F*(2; 18) = 13.35; *p* < 0.0003]. Comparing the two models of visual anticipation over the locomotion, an effect of interaction between the category factor and the visuo-motor condition is also present [*F*(14; 168) = 19.88; *p* < 0.00001]. The distribution of large and small radii of curvatures is not the same whether the subjects use a “non-human” or “human-like” implemented model. Thus, the largest spike is significantly higher in the “human-like” than in the “non-human” condition [*F*(1; 12) = 21.10; *p* < 0.0006], whereas the single rotations are statistically more numerous in the “non-human” than in the “human-like” condition [*F*(1; 12) = 5.63; *p* < 0.04]. The differences between the path patterns are evident if we visually compare two typical exemplars of the recorded robot’s trajectories following the experimental condition (Figure 5).

**Figure 7:**
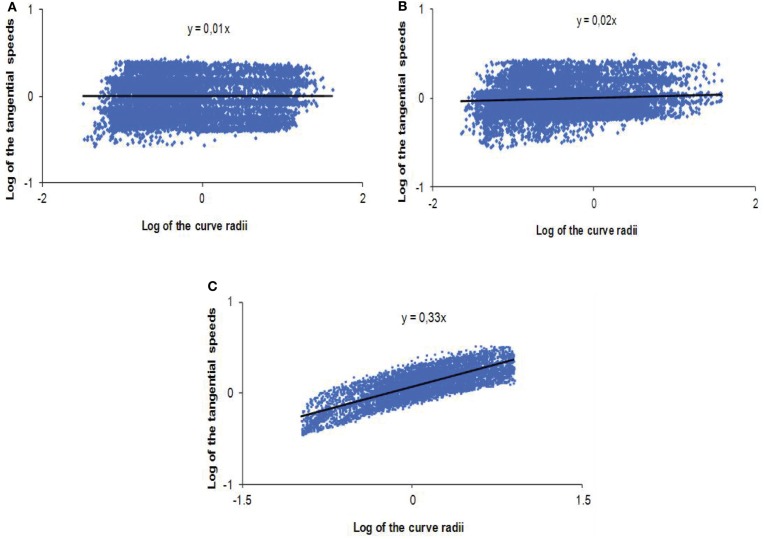
**Logarithmic and standardized representations of the relationship between curve radii and tangential velocities for the unit of the tests in (A) the “control condition,” (B) the “non-human model” of anticipation, and (C) the “human-like model” of anticipation**.

The analysis of the previous parameter shows that when the robot exhibits a visuo-motor coupling strictly identical to the natural human scheme, the participant maximizes the trajectory’s smoothness of the remotely controlled artifact. This tendency to minimize the jerk is a feature that the human being generalizes to the majority of his/her member movements, certainly in order to optimize cost functions (Viviani and Flash, 1995; Todorov and Jordan, 1998; Berret et al., 2011). It seems that this optimization is not only limited to the geometrical characteristics of the trajectory but is also implicated in the relationship between the geometry (curve radii) and the kinematic (linear speed) of the movement. Thus, with the aim of analyzing the instantaneous locomotor behavior through the robot, the tangential velocity for each radius of curvature of the various trajectories was computed. After a logarithmic transformation, the correlation coefficient as well as the slope of the regression line between these two values was analyzed statistically. Then, the curve radii and tangential velocities were standardized for each test and represented according to the experimental condition (Figure 7). To deduce that the teleoperated condition follows the “2/3 Power Law,” the correlation analysis between curve radii and tangential speeds must exhibit a linear relationship with a coefficient of 1/3, if the two variables are plotted according to a logarithmic scale. Otherwise, the behavior cannot be considered as respecting this biological law.

The results show that the “control condition” and the “non-human model” of anticipation do not exhibit a significant correlation between the tangential speeds and the curve radii (*R* = 0.13; NS, for the “control condition” and *R* = 0.16; NS, for the “non-human condition”). Moreover, if we plot the most representative regression line of the radii/velocities logarithmic relationship, we notice that the coefficient is far from the expected ratio of 1/3 (*t* = 29.86; *p* < 0.0001 for DOF = 6, in the “control condition” and *t* = 7.02; *p* < 0.0004 for DOF = 6, in the “non-human condition”), showing that it is near of a value of zero (Figures 7A,B). On the contrary, when the subject controls a robot endowed with a “human-like” visual anticipation over the locomotion, the tangential velocities of the displacement and curve radii are significantly correlated (*R* = 0.76; *p* < 0.001). Figure 7C shows a proportional increase of velocity as the radius gets higher. What is most remarkable is that the regression coefficient of the dot pattern is not statistically different from the “Power Law” ratio of 1/3, for the unit of the trials in this condition (*t* = 0.12; NS for DOF = 6).

To complement the results, an evaluation of the percentage of occurrence of collision was also carried out. The statistical analysis shows that the number of collisions is significantly different between the three conditions [*F*(2; 18) = 4.10; *p* < 0.03]. However, a pairwise comparison does not demonstrate a significant difference between the collision occurrences in the “control condition” vs. the “non-human model” [*F*(1; 12) = 4.32; NS]. Whereas the pairwise comparison between the “control condition” and the “human-like model” points out that the human-like visuo-motor anticipation allows a safer control of the robot, since less collisions occur in this case [*F*(1; 12) = 9.82; *p* < 0.01]. This performance is confirmed by the participants’ subjective perception. The subjects that drive under the “control condition” report a discomfort mainly related to the fact they have a “restricted and too rigid visual field.” In the “non-human condition” the main complaint is about the sensation that “the robot does not seem to be completely under the control of the user.” On the contrary, the “human-like condition” is reported to be “pretty natural” and some individuals even describe this situation in terms of telepresence such as a “distal attribution of themselves” (Loomis, 1992).

## Discussion

This study investigated the consequences of the visuo-locomotor coupling on the prediction of the unfolding steering trajectory. The results show that only a visuo-locomotor coupling temporally organized from the visual sensor to the locomotor organs, allows a remote controlled robot to perform (i) regularly smoothed trajectories and (ii) movement kinematics similar to the ones of the human being. The explanation for the advantage of the head-feet coupling, regarding the opposite coupling, seems to be related to the function of a stabilized referential frame exercised by this segment that supports the visual information. When we walk, the head’s rotation is stabilized around positions that are determined by the direction of the gaze. Consequently, for the brain, the head constitutes a referential platform from which the movements are coordinated (Pozzo et al., 1990). In the case of the “human-like model,” the individual has the possibility to apply a direct control on the orientation of the visual organ. Thus, it is relatively easy for him/her to stabilize the visual referential toward the intended direction, in order to follow up the changes of direction with the minimum jerk. On the contrary, in the “non-human model,” because the camera is not directly controlled, the visual initialization of the displacement cannot be stabilized by the individual, which could explain the discoordination of the ongoing movement. The consequence of this disorder is a sharp pattern of the robot’s displacement. The differences of behavioral performances between the two main experimental conditions are confirmed by the driving accuracy and the subjective feeling of telepresence, which is only reported in the “human-like condition.”

Thus, the main purpose of anticipatory head-orienting strategies is to provide a change of the reference frame by acquiring, in advance, information about the environment in the new direction of heading. From this assumption, we can understand why a visuo-locomotor coupling initiated at the level of the head will provide better predictive information about the unfolding movement parameterization than the converse coupling. Furthermore, according to Viviani and Flash (1995), the proportional relationship between velocity and curvature, described by the “2/3 Power Law,” implies a movement prediction. More precisely, these authors underline that the estimation of the trajectory geometry must be accessible to the motor control system as a part of the internal representation of the predicted movement’s intention. These prediction requirements may explain why only the “human-like model” of anticipation allows the subjects to perform a movement kinematics that respects the “2/3 Power Law.”

This main feature of the temporal organization of the cephalo-pelvic coordination is supported by works carried out on the locomotion guidance during blind walking (Grasso et al., 1996, 1998). These studies show that the head’s anticipation regarding the rest of the body is still present in the darkness. It suggests that the anticipatory movement would be embedded in the motor patterns for curvilinear locomotion and it would not be interpretable on the basis of a unique bottom-up model of simple perception of the optical flow. Thus, this head-body synergy seems indissociable from a general internal model of the action organization (Prévost et al., 2002). It may explain the fact that a transformation of the visuo-locomotor coupling alters the steering kinematics. In addition, because the teleoperated situation of the participants prevents any proprioceptive and vestibular feedback, a trajectory prediction based on an efferent copy of the motor program constitutes the most plausible forward model to interpret the experimental results (Schubotz, 2007).

To summarize, the anticipatory orientation would allow the achievement of a stable frame of reference in time to effectively program and execute an action. This is fundamental because a new direction in locomotion needs to be programmed one step ahead in order to overcome the delays due to biomechanical inertia (Patla et al., 1991). This principle also seems involved in the motor control mediated by an artifact, which means this coordination is representative of a general scheme of the action organization. This is confirmed by the replication of the “2/3 Power Law” through a robot with quite different mechanics in relation to the human being, which brings an interpretation of this law not in terms of peripheral biomechanics factors (Wann et al., 1988; Gribble and Ostry, 1996), but as issued from a internal model of the movement planning (Lacquaniti et al., 1983; Massey et al., 1992). The overall deductions suggest that the “2/3 Power Law” and the visuo-locomotor anticipation are both conditioned by the same internal model of the organization of the movement. The proof is that a perturbation of the temporal organization between the cephalic and the locomotor segments destroys the natural proportionality that exists between the velocity and the geometry of the human movement. Also, the fact that a smooth path and a “2/3 Power Law” are only observed when the visuo-locomotor coupling is implemented according to the human-like behavior is consistent with the theory of an anticipatory construction of the trajectory’s internal model. This mental representation used by the brain as a forward prediction of the path formation seems conditioned by the motor program.

The effect of the visuo-locomotor scheme on the quality of predictive coding of the displacement trajectory, demonstrated in this study, is supported by works that stress the influence of motor competencies on the perception and prediction of the perceived action (Bosbach and Prinz, 2007, for reviews). For instance, when observers have to judge the most uniform motion along an elliptical path, they select the movements that follows the “2/3 Power Law,” showing that the motion perception is mostly constrained by the motor properties (Viviani and Stucchi, 1992). Knowledge of the kinematic laws underlying human movement also seems to inform visual anticipation of the unfolding course of a moving object (Flach et al., 2004; Pozzo et al., 2006). Estimations of the final position of a moving object are more accurate when the movement follows a biological velocity profile rather than a non-biological one. A recent study shows that the famous “Fitts’ Law,” which refers to the relationship between speed and accuracy in produced movements, also holds for action perception (Grosjean et al., 2007). In the same way, works on the perception of biological motion highlight that the biomechanical compatibility of the perceived movement influences the discrimination of motion parameters such as the velocity (Jacobs et al., 2004) or the trajectory geometry (Shiffrar and Freyd, 1990). In the present experiment, the reversion of the visuo-locomotor coupling also consists in a biomechanical alteration of the movement that would distort the perceptual prediction of the steering trajectory, according to the same theory of matching between the participant’s motor knowledge and the ongoing behavior of the robot.

In conclusion, the present findings are in line with previous evidence showing that the motor program of the human body can be mapped isomorphically onto external information about another body, by generating top-down expectations and predictions on its deployment in time (Wilson and Knoblich, 2005; Urgesi et al., 2010; Springer et al., 2011). Mirror neurons, and more specifically the *action observation network* (AON), seem to be involved in this process (Rizzolatti and Sinigaglia, 2010). In fact, several neuroimaging studies have shown that the activation of the mirror neuron system areas is modulated by the observer’s motor experience (Calvo-Merino et al., 2005, 2006; Cross et al., 2006, 2009). According to predictive coding, the optimal state is a minimal prediction error at all levels of the AON, which is achieved when the observed actions match predicted actions (based on prior visuo-motor experience) as closely as possible (Neal and Kilner, 2010; Schippers and Keysers, 2011). This it may explain why the unfamiliar observed coupling between camera and platform results in greater prediction error, while the familiar observed motor scheme would generate a more efficient predictive steering.

Future works on the predictive coding of the steering trajectory will address the question of what happens if the “human-like model,” implemented on the robot, must face more uncertainty. Here, even if the participants completed the experimental path for the very first time, they know what kind of trajectory was expected in each part of the pathway. From an ecological point of view, in order to confirm that the human-like implementation brings a real advantage in terms of anticipatory trajectory planning, this visuo-locomotor coupling should be evaluated when a last-minute change occurs. For instance, the robot could suddenly negotiate a complex ground terrain or face an unexpected obstacle on the road. Such unpredictable events would enable to test the plasticity of our model, regarding the predicted trajectory and motor plan in order to be rapidly modified. When walking on a complex ground surface the fixations of the gaze are directed to regions that the individuals will eventually step onto (Marigold and Patla, 2007). This suggests that task requirements dictate where to fixate and that the following visual guidance of locomotion is achieved according to the “I go where I look” strategy. This tight coupling between fixations and task-relevant surfaces also infers that top-down processes are initiated to detect unexpected events such as potential collisions (Jovancevic et al., 2006). For this to be effective, human beings must execute an appropriate visuo-motor scheme that enhances a proactive behavior to tackle the environmental uncertainty. These arguments, which are consistent with the results of the present study, allow the expectation that the efficiency of “human-like model” could be preserved even in the presence of unpredictable situations.

## Conflict of Interest Statement

The authors declare that the research was conducted in the absence of any commercial or financial relationships that could be construed as a potential conflict of interest.

## References

[B1] Berret B. Chiovetto E. Nori F. Pozzo T. (2011). Evidence for composite cost functions in arm movement planning: an inverse optimal control approach. PLoS Comput. Biol. 7, e1002183 10.1371/journal.pcbi.1002183 22022242 PMC3192804

[B2] Berthoz A. (1997). Le Sens du Mouvement. Paris: Odile Jacob

[B3] Bosbach S. Prinz W. (2007). Perceptual resonance: action-induced modulation of perception. Trends Cogn. Sci. (Regul. Ed.) 11, 349–355 10.1016/j.tics.2007.06.005 17629544

[B4] Calvo-Merino B. Glaser D. E. Grèzes J. Passingham R. E. Haggard P. (2005). Action observation and acquired motor skills: an FMRI study with expert dancers. Cereb. Cortex 15, 1243–1249 10.1093/cercor/bhi007 15616133

[B5] Calvo-Merino B. Grèzes J. Glaser D. E. Passingham R. E. Haggard P. (2006). Seeing or doing? Influence of visual and motor familiarity in action observation. Curr. Biol. 16, 1905–1910 10.1016/j.cub.2006.10.065 17027486

[B6] Cardinali L. Frassinetti F. Brozzoli C. Urquizar C. Roy A. C. Farnè A. (2009). Tool use induces morphological updating of the body schema. Curr. Biol. 19, 478–479 10.1016/j.cub.2009.06.048 19549491

[B7] Carlson T. Alvarez A. Wu D. Verstraten F. (2010). Rapid assimilation of external objects into the body schema. Psychol. Sci. 21, 1000–1005 10.1177/0956797610371962 20483818

[B8] Chattington M. Wilson M. Ashford D. Marple-Horvat D. E. (2007). Eye-steering coordination in natural driving. Exp. Brain Res. 180, 1–14 10.1007/s00221-006-0839-2 17256168

[B9] Cross E. S. Hamilton A. F. D. C. Grafton S. T. (2006). Building a motor simulation de novo: observation of dance by dancers. Neuroimage 31, 1257–1267 10.1016/j.neuroimage.2006.01.033 16530429 PMC1821082

[B10] Cross E. S. Kraemer D. J. M. Hamilton A. F. D. C. Kelley W. M. Grafton S. T. (2009). Sensitivity of the action observation network to physical and observational learning. Cereb. Cortex 19, 315–326 10.1093/cercor/bhn083 18515297 PMC2638791

[B11] Flach R. Knoblich G. Prinz W. (2004). The two-thirds power law in motion perception: when do motor anticipations come into play? Vis. Cogn. 11, 461–481 10.1080/13506280344000392

[B12] Flanagan J. R. Johansson R. S. (2003). Action plans used in action observation. Nature 424, 769–771 10.1038/nature01861 12917683

[B13] Friston K. Mattout J. Kilner J. (2011). Action understanding and active inference. Biol. Cybern. 104, 137–160 10.1007/s00422-011-0424-z 21327826 PMC3491875

[B14] Grasso R. Glasauer S. Takei Y. Berthoz A. (1996). The predictive brain: anticipatory control of head direction for the steering of locomotion. Neuroreport 7, 1170–1174 10.1097/00001756-199604260-00015 8817526

[B15] Grasso R. Prévost P. Ivanenko Y. P. Berthoz A. (1998). Eye-head coordination for the steering of locomotion in humans: an anticipatory synergy. Neurosci. Lett. 253, 115–118 10.1016/S0304-3940(98)00625-9 9774163

[B16] Gribble P. L. Ostry D. J. (1996). Origins of the power law relation between movement velocity and curvature: modeling the effects of muscle mechanics and limb dynamics. J. Neurophysiol. 76, 2853–2860 8930238 10.1152/jn.1996.76.5.2853

[B17] Grosjean M. Shiffrar M. Knoblich G. (2007). Fitts’ law holds for action perception. Psychol. Sci. 18, 95–99 10.1111/j.1467-9280.2007.01854.x 17425525

[B18] Hollands M. A. Patla A. E. Vickers J. N. (2002). Look where you’re going!: gaze behaviour associated with maintaining and changing the direction of locomotion. Exp. Brain Res. 143, 221–230 10.1007/s00221-001-0983-7 11880898

[B19] Iriki A. Tanaka M. Iwamura Y. (1996). Coding of modified body schema during tool use by macaque postcentral neurons. Neuroreport 7, 2325–2330 10.1097/00001756-199610020-00010 8951846

[B20] Iriki A. Tanaka M. Obayashi S. Iwamura Y. (2001). Self-images in the video monitor coded by monkeys intraparietal neurons. Neurosci. Res. 40, 163–173 10.1016/S0168-0102(01)00225-5 11377755

[B21] Jacobs A. Pinto J. Shiffrar M. (2004). Experience, context, and the visual perception of human movement. J. Exp. Psychol. Hum. Percept. Perform. 30, 822–835 10.1037/0096-1523.30.5.822 15462623

[B22] Johansson R. S. Westling G. Bäckström A. Flanagan J. R. (2001). Eye-hand coordination in object manipulation. J. Neurosci. 21, 6917–6932 11517279 10.1523/JNEUROSCI.21-17-06917.2001 PMC6763066

[B23] Jovancevic J. Sullivan B. Hayhoe M. (2006). Control of attention and gaze in complex environments. J. Vis. 6, 1431–1450 10.1167/6.12.9 17209746

[B24] Kandil F. I. Rotter A. Lappe M. (2009). Driving is smoother and more stable when using the tangent point. J. Vis. 9, 1–11 10.1167/9.2.1 19271881

[B25] Lacquaniti F. Maioli C. (1989). The role of preparation in tuning anticipatory and reflex responses during catching. J. Neurosci. 9, 134–148 2913200 10.1523/JNEUROSCI.09-01-00134.1989 PMC6570012

[B26] Lacquaniti F. Terzuolo C. Viviani P. (1983). The law relating the kinematic and figural aspects of drawing movements. Acta Psychol. (Amst.) 54, 115–130 10.1016/0001-6918(83)90027-6 6666647

[B27] Land M. F. (1998). “The visual control of steering,” in Vision and Action, eds Harris L. R. Jenkin K. (Cambridge: Cambridge University Press), 163–180

[B28] Land M. F. Lee D. N. (1994). Where we look when we steer. Nature 369, 742–744 10.1038/369742a0 8008066

[B29] Land M. F. McLeod P. (2000). From eye movements to actions: how batsmen hit the ball. Nat. Neurosci. 3, 1340–1345 10.1038/81887 11100157

[B30] Land M. F. Mennie N. Rusted J. (1999). The roles of vision and eye movements in the control of activities of daily living. Perception 28, 1311–1328 10.1068/p2935 10755142

[B31] Land M. F. Tatler B. W. (2001). Steering with the head: the visual strategy of a racing driver. Curr. Biol. 11, 1215–1220 10.1016/S0960-9822(01)00351-7 11516955

[B32] Loomis J. M. (1992). Distal attribution and presence. Presence (Camb.) 1, 113–118

[B33] Maravita A. Iriki A. (2004). Tools for the body (schema). Trends Cogn. Sci. (Regul. Ed.) 8, 79–86 10.1016/j.tics.2003.12.008 15588812

[B34] Marigold D. S. Patla A. E. (2007). Gaze fixation patterns for negotiating complex ground terrain. Neuroscience 144, 302–313 10.1016/j.neuroscience.2006.09.006 17055177

[B35] Massey J. T. Lurito J. T. Pellizzer G. Georgopoulos A. P. (1992). Three-dimensional drawings in isometric conditions: relation between geometry and kinematics. Exp. Brain Res. 88, 685–690 10.1007/BF00228198 1587327

[B36] Neal A. Kilner J. M. (2010). What is simulated in the action observation network when we observe actions? Eur. J. Neurosci. 32, 1765–1770 10.1111/j.1460-9568.2010.07435.x 20958797 PMC3389780

[B37] Patla A. E. Prentice S. D. Robinson C. Neufeld J. (1991). Visual control of locomotion: strategies for changing direction and for going over obstacles. J. Exp. Psychol. Hum. Percept. Perform. 17, 603–634 10.1037/0096-1523.17.3.603 1834781

[B38] Péruch P. Mestre D. (1999). Between desktop and head immersion: functional visual field during vehicle control and navigation in virtual environments. Presence (Camb.) 8, 54–64 10.1162/105474699566044

[B39] Pozzo T. Berthoz A. Lefort L. (1990). Head stabilization during various locomotor tasks in humans. I. Normal subjects. Exp. Brain Res. 82, 97–106 10.1007/BF00230842 2257917

[B40] Pozzo T. Papaxanthis C. Petit J. L. Schweighofer N. Stucchi N. (2006). Kinematic features of movement tunes perception and action coupling. Behav. Brain Res. 169, 75–82 10.1016/j.bbr.2005.12.005 16430976

[B41] Prévost P. Ivanenko Y. Grasso R. Berthoz A. (2002). Spatial invariance in anticipatory orienting behaviour during human navigation. Neurosci. Lett. 339, 243–247 10.1016/S0304-3940(02)01390-3 12633898

[B42] Reina G. A. Schwartz A. B. (2003). Eye-hand coupling during closed-loop drawing: evidence of shared motor planning. Hum. Mov. Sci. 22, 137–152 10.1016/S0167-9457(02)00156-2 12667746

[B43] Rizzolatti G. Sinigaglia C. (2010). The functional role of the parieto-frontal mirror circuit: interpretations and misinterpretations. Nat. Rev. Neurosci. 11, 264–274 10.1038/nrn2805 20216547

[B44] Rybarczyk Y. Ait Aider O. Hoppenot P. Colle E. (2002). Remote control of a biometrics robot assistance system for disabled persons. AMSE Model. Meas. Control 63, 47–56

[B45] Rybarczyk Y. Hoppenot P. Colle E. Mestre D. (2012). “Sensori-motor appropriation of an artifact: a neuroscientific approach,” in Human Machine Interaction – Getting Closer, ed. Inaki M. (INTECH publisher), 187–212

[B46] Rybarczyk Y. Mestre D. R. (2011). Body schema deformation in teleoperation: effects of sensori-motor contingences. Psychol. Res. 1, 26–41

[B47] Schippers M. B. Keysers C. (2011). Mapping the flow of information within the putative mirror neuron system during gesture observation. Neuroimage 57, 37–44 10.1016/j.neuroimage.2011.02.008 21316466

[B48] Schubotz R. I. (2007). Prediction of external events with our motor system: towards a new framework. Trends Cogn. Sci. (Regul. Ed.) 11, 211–218 10.1016/j.tics.2007.02.006 17383218

[B49] Sebanz N. Knoblich G. (2009). Prediction in joint action: what, when, and where. Top. Cogn. Sci. 1, 353–367 10.1111/j.1756-8765.2009.01024.x 25164938

[B50] Shiffrar M. Freyd J. J. (1990). Apparent motion of the human body. Psychol. Sci. 1, 257–264 10.1111/j.1467-9280.1990.tb00210.x

[B51] Springer A. Brandstädter S. Liepelt R. Birngruber T. Giese M. Mechsner F. Prinz W. (2011). Motor execution affects action prediction. Brain Cogn. 76, 26–36 10.1016/j.bandc.2011.03.007 21477908

[B52] Todorov E. Jordan M. I. (1998). Smoothness maximization along a predefined path accurately predicts the speed profiles of complex arm movements. J. Neurophysiol. 80, 696–714 9705462 10.1152/jn.1998.80.2.696

[B53] Urgesi C. Maieron M. Avenanti A. Tidoni E. Fabbro F. Aglioti S. M. (2010). Simulating the future of actions in the human corticospinal system. Cereb. Cortex 20, 2511–2521 10.1093/cercor/bhp292 20051359

[B54] Vieilledent S. Kerlirzin Y. Dalbera S. Berthoz A. (2001). Relationship between velocity and curvature of a human locomotor trajectory. Neurosci. Lett. 305, 65–69 10.1016/S0304-3940(01)01798-0 11356309

[B55] Viviani P. Cenzato M. (1985). Segmentation and coupling in complex movements. J. Exp. Psychol. Hum. Percept. Perform. 21, 32–53 2934511 10.1037//0096-1523.11.6.828

[B56] Viviani P. Flash T. (1995). Minimum-jerk, two-thirds power law, and isochrony: converging approaches to movement planning. J. Exp. Psychol. Hum. Percept. Perform. 21, 32–53 10.1037/0096-1523.21.1.32 7707032

[B57] Viviani P. Schneider R. (1991). A developmental study of the relationship between geometry and kinematics in drawing movements. J. Exp. Psychol. Hum. Percept. Perform. 17, 198–218 10.1037/0096-1523.17.1.198 1826312

[B58] Viviani P. Stucchi N. (1992). Biological movements look uniform: evidence of motor-perceptual interactions. J. Exp. Psychol. Hum. Percept. Perform. 18, 603–623 10.1037/0096-1523.18.3.603 1500865

[B59] Wann J. P. Nimmo-Smith I. Wing A. M. (1988). Relation between velocity and curvature in movement: equivalence and divergence between a power law and minimum-jerk model. J. Exp. Psychol. Hum. Percept. Perform. 14, 622–637 10.1037/0096-1523.14.4.622 2974873

[B60] Wilson M. Knoblich G. (2005). The case for motor involvement in perceiving conspecifics. Psychol. Bull. 131, 460–473 10.1037/0033-2909.131.3.460 15869341

[B61] Yasui S. Young L. R. (1975). Perceived visual motion as effective stimulus to pursuit eye movement system. Science 190, 906–908 10.1126/science.1188373 1188373

